# Experiences of healthcare, including palliative care, of children with life-limiting and life-threatening conditions and their families: a longitudinal qualitative investigation

**DOI:** 10.1136/archdischild-2020-320189

**Published:** 2020-11-16

**Authors:** Sarah Mitchell, Anne-Marie Slowther, Jane Coad, Jeremy Dale

**Affiliations:** 1 Academic Unit of Primary Care, University of Warwick, Coventry, UK; 2 Department of Oncology and Metabolism, The University of Sheffield, Sheffield, UK; 3 Warwick Medical School, University of Warwick, Coventry, Warwickshire, UK; 4 School of Health Sciences, University of Nottingham, Nottingham, UK

**Keywords:** palliative care, qualitative research, health services research, mortality

## Abstract

**Objectives:**

To understand the experiences and perceptions of healthcare services of children with life-limiting and life-threatening conditions and their family members, including palliative care.

**Design:**

Longitudinal qualitative interview study with children and their family members. Up to three in-depth interviews were conducted over 13 months with each child and family. Data were analysed using thematic analysis.

**Setting:**

Community and hospital settings in the West Midlands, UK.

**Participants:**

Children with a diverse range of life-limiting and life-threatening conditions, aged between 5 and 18 years, and their family members.

**Findings:**

31 participants from 14 families including 10 children took part in 41 interviews. Two children died during the course of the study. Children accepted their conditions as part of life and had other priorities for living. Experiences of ‘fighting’ a fragmented healthcare system that focused on the biomedical aspects of their care were described. The possibility of death was rarely openly discussed. Palliative care tended to be conceptualised as a distinct service or phase of a child’s condition, rather than a broad approach. Access to palliative care depended on the availability of specialist services, and on trusted interpersonal relationships with healthcare professionals who could share uncertainty and the family’s emotional burden.

**Conclusions:**

There is an urgent need to create a more child and family centred approach that enables palliative care to be truly integrated into the wider healthcare of children with life-limiting and life-threatening conditions. Trusted, interpersonal relationships with healthcare professionals, and more effective coordination of care are fundamental to achieving this, and should be valued and enabled throughout the healthcare system.

**What is already known on this topic?**

- Palliative care is an approach to care that is advocated for children living with life-limiting and life-threatening conditions and their families.
- Specialist paediatric palliative care services are associated with benefits including improved symptom control, a feeling of support for families and few intensive treatments towards the end of life.
- Specialist paediatric palliative care services are inconsistently funded and delivered in the UK and internationally.

**What this study adds?**

- The uncertainty and fragility associated with life-limiting and life-threatening conditions in children is rarely addressed openly in a biomedically focused model of care, which presents a significant barrier to the provision of palliative care.
- A key enabler to palliative care is trusted relationships with healthcare professionals; future policy should place greater value on these relationships and the time required to develop them.
- Child and family experiences should inform the development of new models of healthcare with less fragmentation between services, and true integration of specialist paediatric palliative care.

## Introduction

The number of children (under 18 years) living with life-limiting conditions (conditions which cannot be cured and will cause premature death) and life-threatening conditions (where curative treatment is possible but may fail) is rising rapidly.^1^ Their diagnoses are diverse and often associated with complex health and care needs.^2 3^ Over half of all children who die have a pre-existing life-limiting or life-threatening condition.^4^ Most children who die do so in hospital,^5^ often following a prolonged stay in an intensive care environment.^6–8^

‘Palliative care’ is advocated in national and international policy as a multidimensional, active process aimed at improving the quality of life of children with any of the four Together for Short Lives categories of life-limiting or life-threatening condition (table 1).^9–11^ A wide range of palliative care services for children exist. Not all are specialist; some vital palliative care is delivered by other professionals including general practitioners, community teams, therapists and general paediatricians. Specialist paediatric palliative care is most often delivered by teams in acute hospitals and children’s hospices. Referrals to specialist paediatric palliative care services, where these are available, often occur very late in the course of a child’s illness, if at all.^12^ The term ‘palliative care’ is frequently associated with dying, or understood to be a distinct specialist service or phase of a child’s illness. These have all been described as barriers to early identification of palliative care need and referrals.^13–15^

**Table 1:** Together for Short Lives categories^10^

Category	Description
1	Life-threatening conditions for which curative treatment may be feasible but can fail. Access to palliative care services may be necessary when treatment fails or during an acute crisis, irrespective of the duration of threat to life. On reaching long-term remission or following successful curative treatment. there is no longer a need for palliative care services. eg, cancer, organ failure.
2	Conditions where premature death is inevitable. There may be long periods of intensive treatment aimed at prolonging life and allowing participation in normal activities, eg, cystic fibrosis, Duchenne muscular dystrophy.
3	Progressive conditions without curative treatment options. Treatment is exclusively palliative and may commonly extend over many years, eg, batten disease, mucopolysaccharidoses.
4	Irreversible but non-progressive conditions causing severe disability, leading to susceptibility to health. Children can have complex healthcare needs, a high risk of an unpredictable life-threatening event or episode, health complications and an increased likelihood of premature death, eg, severe cerebral palsy, multiple disabilities, such as following brain or spinal cord injury.

Little previous research specifically explores the views and experiences of children with life-limiting and life-threatening conditions, and their family members, in relation to the healthcare that they receive, including palliative care.^16–18^ This study aimed to address that gap.

## Methods

Qualitative research methods were most appropriate for this in-depth exploration of the views and perceptions of children and their families, with longitudinal interviews as the data collection method of choice.^19^ Benefits included opportunities for rapport building and observation of changing needs and experiences of healthcare over time. Interviews allowed for subtle and nuanced aspects of communication to be observed that would be lost through other research methods. Furthermore, the interview process could be tailor made to the needs of each child and family, who could choose the location of and time intervals between the interviews.

### Patient and public involvement

A patient and public involvement group of children and young people aged 9–25 years, including young people with life-limiting conditions and siblings, provided advice throughout the study, from the study design and objectives to the format of interviews for children and dissemination activities.

### Study setting

Children’s hospital and community services in the West Midlands, UK.

### Recruitment

Children aged 5–18 years (school age children) and family members were recruited between October 2016 and June 2017, either by direct invitation from their specialist clinical team, or via leaflets and posters displayed in public areas in the hospital. Any child who met the inclusion criteria (table 2) could take part, regardless of whether they were known to palliative care services. Neonates, preschool children and young people aged over the age of 18 years were excluded. The recruitment target was 14 children and families, with the aim of retaining at least 12 in the study for follow-up interviews.

**Table 2:** Inclusion and exclusion criteria for child and family interviews

Inclusion criteria	1 Children aged 5–18 years (school age) with a life-limiting or life-threatening condition who are under the care of the Community Children’s Nursing Team and/or the Children’s Hospital and who either: receive palliative care services; are aware of (have had discussions about) palliative care services; are living with relapsing or refractory disease; or have had a life-threatening episode (admission to the paediatric intensive care unit). 2 Their family members, who live in the same household.
Exclusion criteria	Children aged <5 years and >18 years. Families of children <5 years and >18 years. Children and families with whom I have clinical contact. Children and/or families who do not wish to participate. Children who are too unwell will not be approached for interview, but their family members may still participate if they wish to. Children who are unable to participate in a conversational interview for any reason related to their condition will not be approached for interview, but their family members may participate if they wish to. Children and families who are unable to provide informed consent in English will not be approached for interview.

### Interview procedures

Child and family interviews were deliberately open and conversational. A topic guide provided structure for the interview but was not prescriptive (table 3).

**Table 3:** Initial topic guide for child and family interviews

For all families	For those aware of ‘palliative care’
**Introduction** **Please tell me your story, in any way that you can/want to** **Please tell me the story of you** *Your story* **Please can you tell me about you?** **Your family?** **Your child(ren**) **What is important to you?** **What do you like to do?** **Which places are important to you?** **Where do you spend your time?** **Which services are involved in your care?** **Who comes to see you?** **What do they do?** What is helpful? What is not? **Which healthcare professionals do you consider to be key in the delivery of your care?** **What works best?** Which services/professionals are most helpful? Which services/professionals do you value most? **What works well? What does not work?** **How do you think services could be improved?** **Do you talk to other children/young people/families about your healthcare/services?** What do you tell your friends? What tends to come up in these discussions? Would you recommend these services to others?	*Palliative care and you (if appropriate)* **Do you have ‘palliative care’ services?** Have you ever heard the term ‘palliative care’? What does that mean to you? What do you receive those services for? What do these services provide for you? Does it matter what a service is called? Do you receive services from the hospice? **Can you tell me how you came to receive palliative care/know the palliative care nursing team/the hospice?** When were you referred? Who brought it up/made the referral? How was this discussed with you? How was that for you/your family? **Do you think that medical/nursing staff receive enough training in this area?** What makes you think that? **Anything else?**

**Figure 1:**
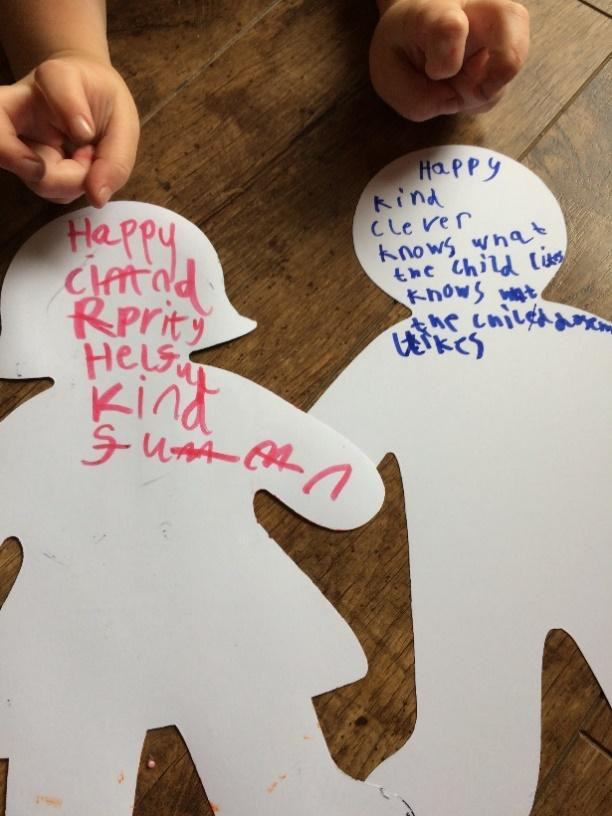
Example of an arts-based activity used during an interview with children.

Interviews were carried out either with the child or family member alone, or together, and in a range of locations including the children’s homes, inpatient wards and outpatient clinics, according to preference and convenience. All interviews were conducted by SM, using passive and active interview techniques, including responding to verbal and non-verbal cues, summarising, reflecting back and silence.^20^ Interviews with children involved a range of age-sensitive techniques such as de-personalising questions, developing a narrative in the third person, using props and toys to encourage story-telling and arts-based activities either as a focus to the interview to facilitate questions (using techniques including draw-write-tell) or as a mutual activity alongside which the interview took place^21–23^ (figure 1).

Interviews ranged in duration from 26 min (with a child) to 108 min (with a mother), median duration was 52 min. Children and their family members would often continue talking after the interview, once the digital recorder was turned off. The further insights that they added were captured in field notes. Children and families were invited to take part in up to three interviews over a 13-month period.

### Data analysis

All interview recordings and field notes were transcribed, anonymised and uploaded into NVivo V.11.^24^ SM led the thematic analysis, which began with familiarisation, reflection and note taking. A description was assigned to every section of interview data, and a series of descriptive codes developed iteratively rather than through the application of a framework.^25^ The codes were grouped into broad overarching conceptual categories, with the emerging codes and concepts being discussed with the supervisory team (JD, A-MS and JC) at monthly intervals, decreasing lone researcher bias.^26^

### Ethical approval

The ethical issues raised by the study are detailed in the research protocol.^19^

### Findings

The first 14 children and families who volunteered for the study were recruited. Thirteen were recruited via their clinical team and one responded to a poster. Parental consent was obtained for every interview. Children could choose whether to sign an agreement form or not.

A total of 41 interviews were conducted with 31 participants from 14 families (10 children, 13 mothers, 6 fathers and 2 brothers). The children had a range of diagnoses and ranged in age from 5 to 18 years (median 9 years). Ten of the families were white British, three were Asian and one was African. Six mothers and two fathers were full-time carers for their children. Six families had experience of a palliative care service. Ten children took part in the interviews, three had little or no verbal communication (C003, C004 and C006) and one (C010) was too unwell to participate. Two of the children died during the course of the research. Data from all interviews was included in the analysis. Details of the study participants and interviews are provided in table 4.

**Table 4:** Overview of study population and interviews

Family participants and identified	Child’s age at recruitment	Male or female	Able to take part in interview?	TfSL category	Number and location of interviews
Child (C001) Mother (M001) Father (F001)	5 Cancer	M	Yes	1	Home Ward Home
Child (C002) Mother (M002) Brother (B002)	17 Congenital	F	Yes	2/3	Home Home Home
Mother (M003) Father (F003)	8 Congenital	F	No (non-verbal communication)	3	Home Home Home
Father (F004)	8 Congenital	F	No (non-verbal communication)	3	1. Home
Child (C006) Mother (M006)	6 Congenital	M	Yes	1	Ward Outpatients Outpatients
Mother (M006)	18 Congenital	M	No (non-verbal communication)	4	Home Home Home
Child (C007) Mother (M007) Father (F007)	7 Cancer	M	Yes	1	Ward Home Home
Child (C008) Mother (M008) Brother (B008)	5 Congenital	M	Yes	1	Home Home Home
Child (C009) Mother (M009) Father (F009)	11 Cancer	F	Yes	1	Outpatients Ward Home
Mother (M010)	5 Congenital	M	No (too unwell)	1/2	1. Ward
Child (C011) Mother (M011) Step-father (F011)	17 Congenital	F	Yes	1	Ward Home Home
Child (C012) Mother (M012)	14 Cancer	M	Yes	1	1. Outpatients
Child (C013) Mother (M013)	14 Cancer	M	Yes	2 (as a result of 1)	Home Home
Child (C014) Mother (M014)	10 Congenital	M	Yes	3	Home Home Home

TfSL, Together for Short Lives.

### Overview of findings

The children who participated tended to divert interviews away from their health and experiences of healthcare to other aspects of their lives, including school, friends and family activities. Children and their family members related to a highly specialist healthcare system, mainly based within a children’s hospital. For some families, the healthcare system also included community teams, a children’s hospice and general practice, but these were not a consistent feature. Four overarching, inter-related themes were identified and are described below.

#### Theme 1: the child does not wish to be defined by their condition

The children did not wish to be defined by their conditions, despite the impact on their health, lifestyle, family, home and personal appearance. ‘*Stay alive, and be happy. That’s the aim’* explained one (C014). A common finding throughout the interviews was that the children would divert the discussion away from healthcare-related topics to other unrelated subjects, ignoring questions about their health and healthcare or declining completely to talk about their medical condition. This often occurred at points where the interview touched on significant moments in their lives, such as a serious deterioration or a hospital admission:

there are times in my life I don’t want to talk about, like (the experience of a cardiac arrest) (Interview 2, C014).

The children wanted to control when they spoke about their condition during the interviews. They had expert knowledge of their conditions, were engaged in their medication regimes and knew when a change in their condition required intervention by a carer or further medical assessment:

Child: Yeah, my hand kept on like going like weird, and then I tried to write and it just kept on going in this funny position every time, the same position and she just… my mum just said, ‘oh it’s nothing’.

SM: Did you think she was wrong, or right?

Child: No, (she was wrong)… because people say like its (problem with electrolytes) all the time, I got cramp and then I was just… it was like in my legs.

Mother: obviously then we took him (to hospital) and they were like, oh my god (there was a diagnosis) (Interview 2, C013 M013)

Despite often diverting discussions away from their condition, they sometimes described feeling that their expert knowledge of themselves and their illness was not recognised by others. They described occasions when their views and concerns were unheard or unaddressed, a situation which could compromise their trust in healthcare professionals. For example, C007 had had to insist to his parents that something was wrong with his health, despite several appointments with his general practitioner, and was diagnosed with cancer following an accident and emergency (A&E) attendance:

M007: We kept thinking ‘why won’t the doctor just give him some antibiotics’, because obviously he’s not getting any better. That carried on and on and on. And then finally he said to us ‘we need to go to the hospital now’. And we took him to A&E then. (Interview 1, M007, C007)

Another example demonstrated how easy it was not to hear the child’s wishes. C009 was seriously unwell at the time of the interview but expressed a desire to go out and play. The request came in the middle of a conversation about his health, and was not acknowledged by any of the adults in the room (including the interviewer):

Mother: … The physios will sort him out when he’s in here. I said while he’s in here and doing nothing they could get him down the gym and that, doing stuff.

Child: Can I play out in a bit? I love that.

Mother: They come up some times and he’s attached to fluid so he’s restricted to go anywhere. But while he’s not he can go down. (Interview 2, M009, C009)

#### Theme 2: the healthcare system can be rigid and fragmented

The interactions of children and family members with a healthcare system that they experienced as rigid, fragmented and disjointed, added to the feeling being unheard and ‘fighting’ to obtain the healthcare that the child needed, as in the quote below:

Mother: It really does my head in, gives me a migraine … I’ve learned from experience that you really have to put yourself out there, if you’re going to sit at home and think ‘oh they’re gonna give it to me, you know bring it to me’, it doesn’t happen … every day is a struggle. (Interview 1, M003)

Referrals to specialist teams occurred frequently, resulting in many different professionals providing care for the child. While expert, specialist care was valued, each referral brought new challenges for the child and family around co-ordination of care, and understanding and assimilating different specialist opinions:

Child: It’s a bit annoying sometimes because there’s so many appointments to go to and it’s all different people.

Mother: It’s different because it’s for each individual different problem isn’t it?

Child: Yeah. (Interview 2, M013, C013)

Children and families also described a biomedical focus on their healthcare that could be informed by the rigid application of guidelines or protocols. This created tension when there was a perception that this did not take into account their child’s individual needs:

Mother: No, they never clarify following a protocol or guidelines. The (clinician) said after a while, it was NHS guidelines. And I was like ‘I know’, but sometimes common sense should be more… you get something in a paper, you’re not going to follow that to a tee, because every child is different … That is just a guide. (Interview 2, M003, F003)

#### Theme 3: trusted interpersonal relationships with healthcare professionals are highly valued

Interpersonal relationships with individual healthcare professionals had a profound effect on how participants experienced care. For the children, healthcare professionals who acknowledged their individual needs, managed procedures with minimal distress, and who were ‘*kind’* were most important. They spoke about clinicians who shared a common interest with them, such as a favourite football team. In the example below, C011, who was in the process of making the transition to adult services, described ‘*brilliant’* healthcare professionals who she had known ‘*since she was little*’:

Child: If I didn’t have to change to any other hospital I’d stay there, because they’ve been absolutely brilliant… I’ve known them since I was little. The two play specialists that I’m seeing … They come up to you to see if you want to do anything. … I would say the nurses are brilliant, because … they give you, like, the right medication and that. (Interview 1, C011, F011)

Family members valued professionals who ‘*really stood up for us’*, ‘*did everything’* and who ‘*used to fight my corner if something was not right’*. Actions and acts of advocacy considered ‘*over and above’* a clinician’s usual role, such as proactively co-ordinating the child’s care, or making themselves accessible via a mobile phone number or email, particularly stood out. There were notable examples of healthcare professionals being alongside families at difficult times:

Mother: He (doctor) came every single morning when [C007] was unwell, every morning, he sat with us and you know that they’ve got other kids to see, and he sat with us… because [C007] was struggling at one point, and of course who does (father) talk to? And so (doctors)’s another man and he just sat with (father) for an hour, just sitting with him, just getting upset also. (Interview 3, M007, C007)

Family members perceived that conflicting demands placed on healthcare professionals compromised their ability to provide this aspect of care:

Mother: I think once they go, especially in hospitals, once they come in that job its ticking boxes. See patient, after patient, after patient, and its lost that caring, the extra is lost down the line. (Interview 2, M003)

Organisational change which led to a change in the healthcare team for the child, such as a change in the way clinics were organised, could be a significant loss. In the example below, a reorganisation of an outpatient clinic would result in the child and family’s care being transferred to a new consultant after several years:

Mother: It’s devastating. It’s as devastating as finding out that she [C008] wasn’t going to live.

…They just keep saying it’s the hospital’s decision… and it was just a conversation that just wasn’t going anywhere… (Interview 1, M008)

#### Theme 4: contemplating the possibility of the child’s death and managing palliative care

Family members described an awareness of the fragility of the child’s life, particularly at times of significant deterioration in their condition. Experiences of discussing this openly both in the interviews and in family interactions with healthcare services were variable. Some had open discussions with well-known and trusted healthcare professionals. Others coped with the possibility of the child dying through denial. Often, there was insufficient time or opportunity to dwell on the possibility that the child may die at the time of an emergency; the life-threatening nature of their child’s condition was only acknowledged after a particular episode had resolved:

Mother: A coping mechanism for me is almost a kind of a, it didn't really happen or it wasn't that bad … And everything points in fact that actually it (the admission to intensive care) was pretty big and pretty bad, but I don't want it to be. … But what am I scared of? You know, acknowledging that it was bad… If I think it’s too bad then I get upset and I'm trying to not get upset. (Interview 2, M001)

For some families, the possibility that their child may die was not a possibility that they could contemplate. They remained focused on medical explanations and solutions, and rejected attempts to discuss palliative care, preferring to focus entirely on new treatment options and onward referrals instead. Furthermore, the word ‘palliative’ was universally unpopular among the children and families in this study. One family specifically requested that ‘the “p” word’ was avoided: ‘*don’t say the “p” word in front of [C002], she doesn’t like it’*. Strong associations with end of life care and dying were a barrier to discussions:

Mother: You'd expect palliative care specialists to be working in a hospice because to me a hospice again is all about that. And I know it’s different for children, but it is sort of about end of life. And I know children they talk more about life-limited and life-threatening don't they, life-threatening rather than life-limited, but yeah. (Interview 2, M006)

‘Palliative care’ was often conceptualised as a distinct and separate service, rather than as a broad, holistic approach to improve quality of life, delivered by a range of healthcare professionals. Relationships with specialist palliative care services were mixed (six of the children received care from these services) as were the services received (ranging from complex symptom control to respite care at a hospice). Some described relying on palliative care professionals for aspects of their care that they struggled to access elsewhere, including specific clinical interventions (such as regular injections) or care coordination. Referrals to palliative care services could be limited by referral criteria. In the example below, the child fluctuated between meeting specific criteria for a palliative care service, and not:

Mother: Yeah, because… because they (the palliative care team) was going to let us go. She didn’t fit the criteria. But then when she got this poorly this time, she fit the criteria again … So you know when she picks up, well she is picking up again. So when she picks up again they’ll probably say ‘no’ again. (Interview 3, M002)

## Discussion

### Summary

This study describes how children with life-limiting and life-threatening conditions and their family members perceive healthcare services, providing insights into their varied experiences of palliative care. The children tended to accept their conditions as part of life, were not always keen to engage in discussions about their conditions and wanted some control over when they talked about their illness. Children and families had an awareness of the fragility of the child’s life, but the possibility of dying was rarely spoken about. The children and families expressed a need for individualised and co-ordinated healthcare, but felt this was lacking due to the organisation of healthcare services into multiple specialties. The ‘fight’ with the system described by every family was sometimes addressed through acts of advocacy from healthcare professionals who knew them well, rather than a coordinated system response.

Specialist palliative care services varied in terms of both the care provided for each individual child and family, and how they were accessed. ‘Palliative care’ tended to be considered a separate specialist service bound by specific referral criteria, rather than being integrated into the personalised care of each child and family.

### Strengths and limitations

A strength of this study was that it included the views and perceptions of children, alongside those of their families. The longitudinal approach allowed for insights into their changing needs and experiences of healthcare services over time.^27^ The development of rapport with children and their families also resulted in them sharing very detailed, in-depth accounts of their experience. Most of the children who contributed did so only at the second and third interviews once they had experienced the interview and built some trust in the researcher.

There was diversity in the study population in terms of age, ethnicity and the child’s condition. The study population was relatively small, and is likely to represent children and families with motivation to participate, so their view may not be representative of a wider population. Children who could not communicate verbally, neonates, preschool children and young people over the age of 18 years making the transition to adult services were beyond the scope of this study, but all warrant further research. Notably, only one child with severe static neurological disability (Together for Short Lives category 4) took part, although many of these children have palliative care needs. Most interviews were conducted with children and their family members. Their differing views were not compared in this study. This would also be valuable future research.

### Comparison with existing literature

As in previous research, the children in this study had a desire to maintain normal life as much as possible.^18 28^ Life with intensive medical treatments, chronic uncertainty and an awareness of the possibility of death, was normal.^29–31^ Trusted, authentic interpersonal relationships with healthcare professionals within which children could be heard, and which allowed families to feel that their emotional burden was shared, enabled open and honest communication about their situation.^32 33^ These required time and consistency which could be compromised due to conflicting demands on individual clinicians and healthcare service organisation.

The finding of parents ‘fighting’ the system to obtain the healthcare that their child needed has been described elsewhere,^18^ and is in stark contrast to the emphasis on choice in palliative care policy documents.^34^ As in previous studies, the word ‘palliative’ was often associated with the end of life and dying by the families in this study.^13 14^ This provided a pertinent barrier to the provision of such care.

### Implications for policy and practice

The design of healthcare services to meet the needs of the increasing numbers of children with life-limiting and life-threatening conditions, and their increasingly complex needs, is a pressing issue. Children and families value trusted interpersonal relationships with healthcare professionals that help them to feel heard in a fragmented healthcare system, and that can enable discussions about the possibility of dying and palliative care. There is evidence of the benefits of specialist paediatric palliative care services for children including improved symptom control, improved quality of life, more care at home if this is the preferred place and a feeling of support for families.^16 17 35^ The findings of this study suggest that currently there is not enough awareness of these benefits, and there is a need to improve understanding of the role of specialist paediatric palliative care services. Simple explanations and information have been shown to make the concept of palliative care more acceptable to children and families.^15 36^ This is an important area for future work, as is the development of new models of healthcare with less fragmentation between services, and true integration of specialist paediatric palliative care into other services. Prioritising trusted relationships provides a good foundation for such models of care.

## Conclusion

Children with life-limiting and life-threatening conditions, and their family members value trusted relationships with healthcare professionals. These relationships are key to the delivery of the proactive, holistic, co-ordinated healthcare that children and families desire, and to the integration of palliative care, as an approach to care or through referral to a specialist service. Future healthcare service delivery and policy in both palliative care and healthcare for children should place more value on these relationships to enable the delivery of palliative care in practice.

## Data Availability

Data are available on reasonable request from the authors.
